# A *rel* *A*‐dependent regulatory cascade for auto‐induction of microbisporicin production in *M* *icrobispora corallina*

**DOI:** 10.1111/mmi.13046

**Published:** 2015-05-29

**Authors:** Lorena T. Fernández‐Martínez, Juan P. Gomez‐Escribano, Mervyn J. Bibb

**Affiliations:** ^1^ Department of Molecular MicrobiologyJohn Innes CentreNorwich Research ParkNorwichNR4 7UHUK

## Abstract

Microbisporicin is a potent type I lantibiotic produced by the rare actinomycete *M* *icrobispora corallina* that is in preclinical trials for the treatment of infections caused by methicillin‐resistant isolates of *S* *taphylococcus aureus* (MRSA). Analysis of the gene cluster for the biosynthesis of microbisporicin, which contains two unique post‐translationally modified residues (5‐chlorotryptophan and 3, 4‐dihydroxyproline), has revealed an unusual regulatory mechanism that involves a pathway‐specific extracytoplasmic function sigma factor (MibX)/anti‐sigma factor (MibW) complex and an additional transcriptional regulator MibR. A model for the regulation of microbisporicin biosynthesis derived from transcriptional, mutational and quantitative reverse transcription polymerase chain reaction analyses suggests that MibR, which contains a C‐terminal DNA‐binding domain found in the LuxR family of transcriptional activators, functions as an essential master regulator to trigger microbisporicin production while MibX and MibW induce feed‐forward biosynthesis and producer immunity. Moreover, we demonstrate that initial expression of *mib* *R*, and thus microbisporicin production, is dependent on the ppGpp synthetase gene (*relA*) of *M* *. corallina*. In addition, we show that constitutive expression of either of the two positively acting regulatory genes, *mib* *R* or *mib* *X*, leads to precocious and enhanced microbisporicin production.

## Introduction

Lantibiotics are ribosomally synthesised, post‐translationally modified peptides with antimicrobial activity that are produced by a number of Gram‐positive bacteria (Li and O'Sullivan, 2012). They contain lanthionine and/or methyl lanthionine bridges, which contribute to their relative resistance to proteolytic cleavage, structural rigidity and target specificity (Chatterjee *et al*., 2005). Only a few lantibiotic biosynthetic gene clusters derived from actinobacteria have been characterised thus far – all of them chromosomally located (Li and O'Sullivan, 2012). These clusters typically contain genes encoding the precursor peptide, enzymes responsible for a variety of posttranslational modifications, proteins involved in export and immunity, and frequently pathway‐specific regulatory proteins (Chatterjee *et al*., 2005; Arnison *et al*., 2013). Microbisporicin is a potent lantibiotic produced by *Microbispora corallina* (Nakajima *et al*., 1999); it is also made by *Microbispora* sp. American Type Culture Collection (ATCC) PTA5024 and known commercially as NAI‐107 (Donadio *et al*., 2009; Jabés *et al*., 2011). It is active against a wide range of Gram‐positive pathogens, including multiply drug resistant *Staphylococcus aureus* strains. Microbisporicin inhibits cell wall biosynthesis by binding to lipid II, the immediate precursor for peptidoglycan biosynthesis (Lazzarini *et al*., 2005; Münch *et al*., 2014). The lantibiotic contains one methyllanthionine and three lanthionine bridges, a S‐[(Z)‐2‐aminovinyl]‐D‐cysteine at its C terminus and two unique modifications, 5‐chlorotryptophan and 3, 4‐dihydroxyproline (Lazzarini *et al*., 2005; Castiglione *et al*., 2008). The absence of these latter two modifications markedly reduces the potency of the compound (Maffioli *et al*., 2014). Previous studies identified a cluster of 20 genes involved in microbisporicin biosynthesis, which to our knowledge, is the largest lantibiotic gene cluster identified thus far (Foulston and Bibb, 2010). It encodes a putative transcriptional activator, MibR, as well as an extracytoplasmic function (ECF) sigma factor and anti‐sigma factor pair, MibX and MibW, respectively, all involved in the regulation of microbisporicin biosynthesis. Foulston and Bibb (2011) suggested a model in which MibR acts as a master regulator to promote low levels of production of the immature, less active form of microbisporicin that lacks the unique modifications referred to earlier; this intermediate then triggers a feed‐forward mechanism mediated by the ECF‐sigma factor MibX that results in high levels of microbisporicin production. However, the direct targets of MibR and MibX remained to be confirmed experimentally, as did the signal triggering the initial activation of MibR transcription.

Antibiotic production in actinomycetes is triggered frequently by nutrient limitation (Bibb, 2005; Martín and Liras, 2012) presumably affording a selective advantage to the producing organism under starvation conditions. Guanosine tetraphosphate (ppGpp) is a key intracellular signalling molecule for sensing nutrient starvation and triggering adaptive responses in a wide range of bacteria. ppGpp induces a rapid response to amino acid starvation in *Escherichia coli*, *Streptomyces* species and other bacteria, reducing the expression of genes involved in rapid growth and often activating transcription of genes involved in specialised metabolism (Takano and Bibb, 1994; Bremer and Ehrenberg, 1995; Ochi, 2007; Gaca *et al*., 2015). Under conditions of nitrogen limitation, the ribosome‐bound RelA synthesises ppGpp in response to uncharged tRNAs that bind to the ribosomal A site (Cashel *et al*., 1996). In *E. coli*, ppGpp elicits transcriptional changes by interacting directly with RNA polymerase (Magnusson *et al*., 2005) while in *Bacillus subtilis*, ppGpp regulation of gene expression appears to be mediated through GTP pool levels, therefore modulating promoter activity indirectly (Krásný and Gourse, 2004). In *Streptomyces coelicolor*, ppGpp synthesis was shown to be required for antibiotic production under conditions of nitrogen limitation (Chakraburtty and Bibb, 1997); moreover, induction of ppGpp synthesis at levels that did not influence growth rate and under conditions of nutritional sufficiency invoked the transcription of the pathway‐specific regulatory gene *act*II‐orf4 and actinorhodin production (Hesketh *et al*., 2001).

In this study, we demonstrate the role of RelA, and presumably ppGpp synthesis, in the activation of microbisporicin biosynthesis by initially triggering the production of a precursor that subsequently induces high levels of production of the mature antibiotic. We also show that the lantibiotic can act as an extracellular signalling molecule to trigger microbisporicin production in the wider *M. corallina* community. We identify the individual targets of MibX and MibR, firmly establishing the complex regulatory cascade that leads to microbisporicin biosynthesis. Finally, we identify an ABC transporter that appears to confer some level of immunity to microbisporicin, and that is also required for production of the lantibiotic.

## Results

### *gusA* transcriptional fusions in *S* *. coelicolor* M1152 verify the targets of MibX and MibR

**Figure 1:**
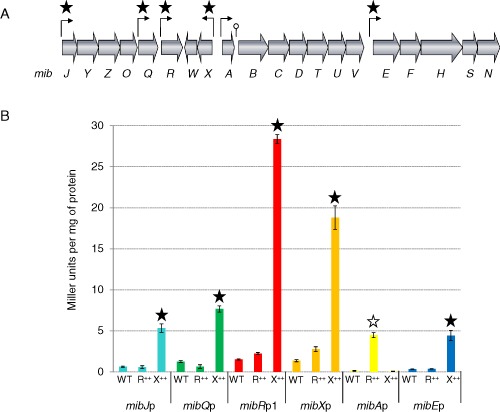
A. The microbisporicin biosynthetic gene cluster (Foulston and Bibb, 2010). Transcriptional start sites are marked by arrows and those promoter regions containing the predicted ECF‐sigma factor consensus sequence are indicated by stars; the loop downstream of *mib* *A* indicates a putative attenuator. B. β‐glucuronidase activity assays of *mib* promoter regions fused to *gus* *A* in *S* *treptomyces coelicolor* M1152 and derivatives containing constitutively expressed *mib* *R* (R++) or *mibX* (X++) were carried out after 72 h of growth. β‐Glucuronidase activity is expressed as Miller units mg^−1^ of protein. The filled stars indicate promoters regulated by MibX and the open star indicates the promoter regulated by MibR.

Microbisporicin production occurs in a growth phase‐dependent manner, commencing towards the end of rapid growth (Foulston, 2010). The microbisporicin biosynthetic gene cluster consists of six operons (Fig. 1A). Previous studies (Foulston and Bibb, 2011) identified an ECF‐sigma factor consensus sequence (GAACC‐N15‐GCTAC) located 8–10 nucleotides upstream of the transcriptional start sites of *mibJ*, *mibQ*, *mibR*, *mibX* and *mibE*, suggesting that the transcription of these genes and operons is activated directly by MibX. In contrast, the promoter region of the crucial *mibABCDTUV* biosynthetic operon lacks this sequence, but contains instead the motif TTGACA‐N17‐TCGACT that is likely to be recognised by the RNA polymerase holoenzyme containing the major vegetative sigma factor of *M. corallina* [the homologue of sigma *hrdB* of *S. coelicolor* (Brown *et al*., 1992; Foulston and Bibb, 2011)]. MibR, which is essential for microbisporicin production, could thus be a candidate for activating the transcription of the *mibA* operon in a growth phase‐dependent manner. To evaluate these bioinformatic predictions, the *mibJ* (299bp), *mibQ* (166 bp), *mibR* (384 bp), *mibX* (254 bp), *mibA* (244 bp) and *mibE* (440 bp) promoter regions were cloned separately upstream of a *Streptomyces* codon‐optimised β‐glucuronidase gene, *gusA*, in pGUS (Myronovskyi *et al*., 2011), and the resulting plasmids (Table 1) integrated into the ΦC31 *attB* sites of *S. coelicolor* M1152 derivatives M1598, M1594 and M1595; the last two strains carried derivatives of pIJ10257 (which integrates at the ΦBT1 *attB* site) in which *mibR* or *mibX*, respectively, were constitutively expressed from ermE*p, while M1598 contained just the vector pIJ10257 (Table 1).

**Table 1:** Plasmids used and constructed in this study

Vector	Description	Reference
pUZ8002	*tra*, *neo*, RP4	J. Wilson and D. Figurski, personal communication
pIJ8600	*oriT*, ΦC31 *attB‐int*, APR^R^, *tipA*p	Takano *et al*., 1995
pIJ12572	pIJ8600 + *mibR*	This work
pIJ10257	*oriT*, ΦBT1 *attB‐int*, HYG^R^, ermE*p	Hong *et al*., 2005
pIJ12574	pIJ10257 + *mibX*	This work
pIJ12576	pIJ10257 + *mibR*	This work
pIJ12743	pIJ10257 + *mibEF*	This work
pIJ12750	pIJ10257 + I‐SceI	This work
pIJ12738	pKC1132 with MCS and I‐SceI site from pUC57‐Simple_SceI	Fernández‐Martínez and Bibb, 2014
pGUS	*oriT*, ΦC31 *attB‐int*, APR^R^, promoterless codon‐optimised *gusA*	Myronovskyi *et al*., 2011
pIJ12579	pGUS + *mibA* promoter region	This work
pIJ12580	pGUS + *mibE* promoter region	This work
pIJ12581	pGUS + *mibJ* promoter region	This work
pIJ12582	pGUS + *mibQ* promoter region	This work
pIJ12583	pGUS + *mibR* whole (including p1 and p2) promoter region	This work
pIJ12584	pGUS + *mibX* promoter region	This work
pIJ12586	pGUS + *mibR*p2 promoter region	This work

APR, apramycin; HYG, hygromycin B.

Only very low levels of *gusA* expression were detected from the six *mib* promoters in the absence of *mibR* and *mibX* (Fig. 1B). However, constitutive expression of *mibX* resulted in marked induction of GusA activity from the *mibJ*, *mibQ*, *mibR*, *mibX* and *mibE* promoters, all of which contain the ECF‐sigma factor consensus sequence, but not from the *mibA* promoter fusion in which the ECF‐sigma factor motif is absent (Fig. 1B, induction indicated by black stars). In contrast, induction of the *mibA* promoter was only detected in the strain containing the constitutively expressed *mibR* (Fig. 1B, indicated by the white star). Thus, in *S. coelicolor*, MibR activates transcription of the *mibA* operon whereas MibX activates transcription of the other five genes and operons in the microbisporicin biosynthetic cluster.

### Analysis of the *mibR* promoter region reveals a second promoter independent of MibX

**Figure 2:**
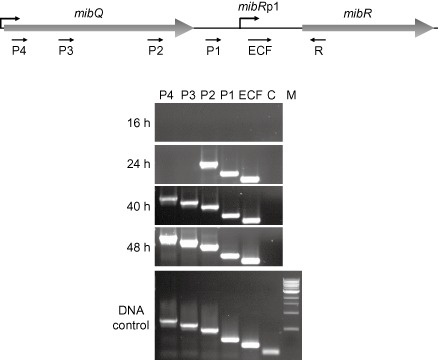
Top: Schematic representation of *mib* *Q*, *mib* *R* and their intergenic region. Bottom: Forward oligonucleotides P4, P3, P2, P1 and ECF were used in pairwise combinations with reverse oligonucleotide R in RT‐PCR reactions to identify the approximate location of the promoter upstream of *mib* *R*p1. RNA was isolated from *M* *icrobispora corallina* grown in VSPA liquid medium at the time points indicated. Genomic DNA was used as a control to confirm the authenticity of each oligonucleotide pair. C represents amplification from an internal *mib* *R* fragment used as a negative control to verify the absence of DNA from the RNA samples. M, the 1Kb NEB ladder (NEB).

The studies described earlier demonstrated that the ECF‐like *mibR* promoter (*mibR*p1) is indeed dependent on the ECF‐sigma factor MibX for its activation. However, earlier S1 nuclease protection analyses had suggested that there was a second *mibR* promoter located further upstream (Foulston and Bibb, 2011) that might also be involved in the activating *mibR* expression. In an attempt to further characterise this putative promoter, reverse transcription polymerase chain reaction (RT‐PCR) experiments were carried out using RNA extracted from *M. corallina* 16, 24, 40 and 48 h after inoculation of production medium using a series of paired and nested oligonucleotide primers covering the sequence upstream of *mibR*p1 (Table S1). This revealed the presence of a second putative promoter element, *mibR*p2, located 449 nt–261 nt upstream of the *mibR* coding sequence that was active 24 h after inoculation, well before transcriptional read‐through from *mibQ* and microbisporicin production was observed (Fig. 2). The putative transcriptional start site of *mibR*p2 was determined using 5′ extension RACE (Fig. S1) and shown to lie 439–440 nt upstream of the *mibR* GTG start codon, inside the *mibQ* coding sequence. Inspection of the nucleotide sequence preceding this putative transcriptional start site failed to reveal any striking similarity to known promoter sequences, including the canonical ECF‐sigma factor recognition motif and consequently it would not be predicted to be activated by MibX.

### Transcriptional activation of *mibR*p2 is *relA*‐dependent

**Figure 3:**
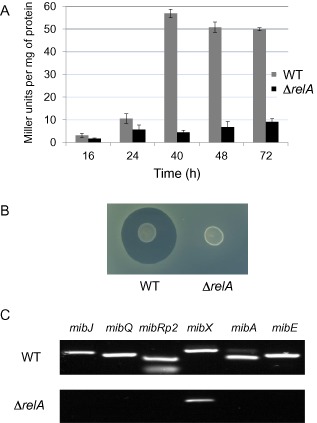
A. *mib* *R*p2‐derived β‐glucuronidase activity in *S* *treptomyces coelicolor* M145 and M571 (Δ*relA*). β‐Glucuronidase activity is given in Miller units mg^−1^ of protein. B. Anti‐microbial activity of the *M* *icrobispora corallina rel* *A* mutant compared with the wild‐type strain (WT). The strains were spotted on SMMS agar medium and incubated at 30°C for 10 days. The plate was then overlaid with a lawn of *M* *icrococcus luteus* and incubated overnight at 30°C before zones of inhibition were visualised. C. RT‐PCR analysis of gene expression in all six of the *mib* operons in the wild‐type and *relA* mutant strains of *M* *. corallina*. The individual genes chosen for analysis are given; *mib* *R*p2 indicates that the amplified region corresponds to sequences present in the transcript initiating at *mib* *R*p2 that are not present in the *mib* *R*p1 transcript.

Previous studies had implicated ppGpp, produced by the ribosome‐bound ppGpp synthetase (RelA), as an intracellular signalling molecule for the initiation of antibiotic production in actinomycetes (Takano and Bibb, 1994; Martínez‐Costa *et al*., 1998; Ochi, 2007). Moreover, deletion of *relA* in *S. coelicolor* abolished the production of both actinorhodin and the undecylprodiginine complex of compounds under conditions of nitrogen limitation (Chakraburtty and Bibb, 1997), while induction of ppGpp synthesis at levels that did not impair growth activated actinorhodin gene transcription (Hesketh *et al*., 2001). To determine whether transcription from *mibR*p2 was dependent on ppGpp, a 478 bp fragment containing *mibR*p2 (corresponding to nt sequence 5245–5723 of GenBank accession HM536998.1) was cloned in pGUS generating pIJ12586, which was then introduced into the ΦC31 *attB* sites of *S. coelicolor* M145 (*relA* ^+^) and *S. coelicolor* M571 (Δ*relA*), and GusA activity assayed throughout growth in nitrogen‐limited Supplemented liquid Minimal Medium (SMM) (Fig. 3A). The results demonstrate that transcription from *mibR*p2 in *S. coelicolor* under these growth conditions is RelA‐ (and presumably ppGpp) dependent.

### Deletion of *relA* in *M* *. corallina* abolishes microbisporicin production

To determine whether microbisporicin production was RelA‐dependent in the natural producer, we adopted the Meganuclease strategy (Fernández‐Martínez and Bibb, 2014) to construct a markerless *relA* deletion mutant of *M. corallina* (see Experimental Procedures). *M. corallina* is a difficult strain to work with; it grows slowly, taking 2–3 weeks to obtain workable colonies and it sporulates poorly, necessitating the use of mycelial fragments for most genetic manipulations. Consequently, the availability of the Meganuclease system that allows the selection of second crossover events during gene deletion by homologous recombination is extremely valuable. The resulting *relA* mutant, M1596, was grown adjacent to the wild‐type strain on nitrogen‐limited Supplemented liquid Minimal Medium Solid (SMMS) agar (Chakraburtty *et al*., 1996). After 10 days of incubation, the strains were overlaid with the sensitive indicator strain *Micrococcus luteus* (Fig. 3B). While wild‐type *M. corallina* showed a clear zone of inhibition, the *relA* mutant was devoid of antibiotic activity. To assess the effect of deletion of *relA* on transcription of the *mib* cluster, RT‐PCR experiments were carried out using RNA extracted from M1596 and from the wild‐type strain 48 h after inoculation in SMM liquid medium using a series of oligonucleotide primers (Foulston and Bibb, 2010) covering regions of each of the six *mib* cluster operons (Fig. 1A). Deletion of *relA* resulted in no detectable transcription from the *mibR*p2 promoter nor of any of the other *mib* operons except for *mibXW*, where transcription appeared to be reduced, but not abolished.

### A model for the regulation of microbisporicin production in *M* *. corallina*

**Figure 4:**
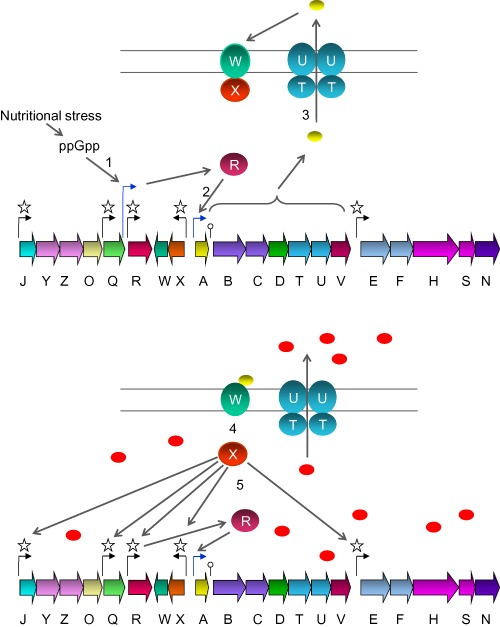
Model for the regulation of microbisporicin biosynthesis. Top: Prior to detectable microbisporicin production, MibW sequesters MibX at the membrane, preventing its interaction with target promoters. ppGpp, induced by nutrient limitation, activates transcription of *mib* *R*p2 (1). MibR then activates transcription of the *mib* *ABCDTUV* operon (2) leading to production of the immature, less active form of microbisporicin (yellow ovals) which is the exported by MibTU (3). Bottom: Interaction of the peptide with MibW, or a low level of inhibition of peptidoglycan biosynthesis that may be perceived by the anti‐sigma factor, results in the release of MibX (4), and high‐level expression of the entire *mib* gene cluster (5) resulting in the formation of the fully modified and active form of microbisporicin (red ovals).

Based on the results presented so far, we propose an update to our previous model (Foulston and Bibb, 2011) for the regulation of microbisporicin production that explains its growth phase‐dependence (Fig. 4). During growth under conditions of nutrient sufficiency, the system is poised for activation with MibX, produced from a basal level of expression, sequestered at the membrane by its cognate anti‐sigma factor MibW (*mibXW* are co‐transcribed) (Foulston and Bibb, 2010; 2011). Under conditions of nitrogen limitation, uncharged tRNAs bind to the ribosomal A‐site, activating the ribosome‐bound RelA resulting in ppGpp synthesis. This then leads to a low level of expression of *mibR* from the *relA*‐dependent *mibR*p2 promoter, and consequently, a low level of transcription of the *mibABCDTUV* operon and the production of a small amount of the immature and less active form of the lantibiotic lacking the chlorination of tryptophan at position 4, and the di‐hydroxylation of proline at position 14 (Lazzarini *et al*., 2005) This less active form of the lantibiotic is exported out of the cell by the ABC transporter MibTU (Foulston and Bibb, 2010) where it could interact either with MibW or its target lipid II resulting in cell envelope stress potentially sensed by MibW. In either case, MibX is released from MibW leading to transcription of all of the MibX‐dependent genes and operons, which include *mibR*, resulting in high levels of *mibR* expression and consequently, high‐level expression of the *mibABCDTUV* operon and production of the mature antibiotic. Thus microbisporicin production, at least under conditions on nitrogen limitation, is triggered by nutrient limitation sensed by RelA; the likely subsequent ppGpp synthesis then leads to a feed‐forward regulatory mechanism that results in high levels of lantibiotic biosynthesis.

### Constitutive expression of *mibR* or *mibX* triggers early and enhanced production of microbisporicin

**Figure 5:**
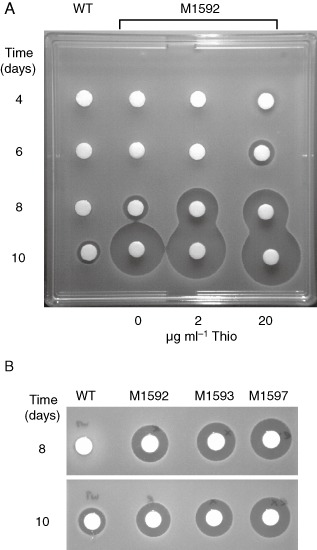
A. Antimicrobial activity of M1592 (*M* *icrobispora corallina* containing *mib* *R* expressed from *tip* *A*p) compared with the wild‐type strain (WT). The strains were grown in VSPA at 30°C and culture supernatants sampled at different time points. M1592 was also grown at two different concentrations of thiostrepton (inducer of *tip* *A*p). Forty microlitre of samples of culture supernatants were applied to filter paper discs, which were dried and placed on a lawn of *M* *icrococcus luteus*. The plate was incubated overnight at 30°C before zones of inhibition were visualised. B. Antimicrobial activity of *M* *. corallina* constitutively expressing *mibR* (M1592), *mibX* (M1593) and both *mibR* and *mibX* simultaneously (M1597) compared with the wild‐type strain (WT). Strains were grown in VSPA liquid medium at 30°C and 40 μl of samples of culture supernatants were applied to filter paper discs which were dried and placed on a lawn of *M* *icrococcus luteus*. The plate was incubated overnight at 30°C before zones of inhibition were visualised.

The data and model presented thus far indicate that both MibR and MibX function as transcriptional activators to trigger microbisporicin biosynthesis. To assess the effect of constitutive expression of *mibX* and *mibR* on microbisporicin production, pIJ12572, containing *mibR* under the control of the thiostrepton‐inducible *tipA* promoter and pIJ12574, containing *mibX* under the control of the constitutive ermE* promoter, were integrated into the ΦC31 and ΦBT1 *attB* sites of *M. corallina*, respectively. Constitutive expression of *mibR* (the *tipA* promoter exhibits basal levels of expression in the absence of thiostrepton; Murakami *et al*., 1989; Ali *et al*., 2002) resulted in precocious and increased levels of microbisporicin production, which were further enhanced in the presence of the inducer (Fig. 5A; confirmed by Matrix‐Assisted Laser Desorption/Ionisation‐Time of Flight (MALDI‐ToF) analysis, data not shown). Constitutive expression of *mibX* also caused precocious microbisporicin production and at much higher levels than in the wild‐type strain (Fig. 5B). Simultaneous constitutive expression of both *mibR* and *mibX* resulted in even higher levels of microbisporicin biosynthesis (Fig. 5B). These results confirm that both MibR and MibX function as positively acting regulators of microbisporicin biosynthesis in *M. corallina*.

### Microbisporicin acts as a signalling molecule in *M* *. corallina* to induce its own production

**Figure 6:**
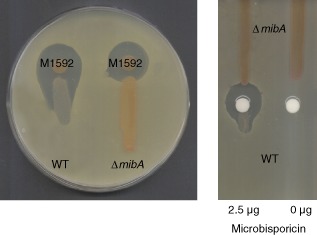
Microbisporicin induces its own production. Left plate: *M* *icrobispora corallina* M1592 constitutively expressing *mibR* and precociously producing the lantibiotic was spotted next to streaks of (left) the wild‐type (WT) strain and (right) a non‐producing Δ*mib* *A* mutant. Right plate: Addition of purified microbisporicin to a filter paper disc triggers precocious microbisporicin biosynthesis in the *M* *. corallina* wild‐type strain, but not in the Δ*mib* *A* mutant. The strains were grown on MV01 agar medium at 30°C for 5 days and then overlaid with a lawn of *M* *icrococcus luteus* in soft nutrient agar. The plates were incubated overnight at 30°C before zones of inhibition were visualised. The inverted pear‐shaped inhibition zones indicate precocious induction of microbisporicin production in the mycelium located proximal to the source of the compound.

The model presented earlier for the regulation of microbisporicin production is essentially an example of auto‐induction, where the production of a small amount of the immature form of the lantibiotic functions in a feed‐forward mechanism initiated by nutrient limitation and ppGpp synthesis to subsequently trigger high levels of production. But in principle, once transported out of the cell, both forms of the lantibiotic could interact with other members of the *M. corallina* community that are not nutrient limited to coordinate microbisporicin production, perhaps in an attempt to achieve ecologically relevant levels of antibiotic activity. To address this possibility, *M. corallina* M1592 (the *mibR* over‐expression strain), which produces microbisporicin precociously, was spotted on V0.1 agar (Marcone *et al*., 2010) plates in close proximity to streaks of the wild‐type *M. corallina* strain and the Δ*mibA* non‐producing mutant M1127 (Foulston and Bibb, 2010). After 5 days of incubation, when the wild‐type strain had not commenced microbisporicin biosynthesis (usually detected from 6 days onwards), the plates were overlaid with soft nutrient agar containing the indicator strain *Micrococcus luteus* (Fig. 6 left plate). The inverted pear‐shaped zone of inhibition revealed induction of microbisporicin biosynthesis in the wild‐type mycelia closest to M1592 while the inhibition zone next to the non‐producer control strain remained circular. To confirm that this induction of production was due to microbisporicin, 2.5 μg of the lantibiotic were spotted onto an antibiotic assay disc next to streaks of wild‐type *M. corallina* and the Δ*mibA* non‐producing mutant. Again, the inverted pear‐shaped inhibition zone indicated autoinduction of microbisporicin production (Fig. 6, right plate) in the wild‐type strain, but not in the *mibA* mutant. A range of other antibiotics was also tested for their ability to induce microbisporicin production, including the cell wall biosynthesis inhibitors vancomycin, planosporicin, fosfomycin, bacitracin, carbenicillin and tunicamycin and the protein synthesis inhibitor apramycin (data not shown). A range of concentrations were used, but none induced precocious microbisporicin production even at concentrations at which some of the antibiotics inhibited growth of *M. corallina*. These results demonstrate that microbisporicin can induce its own synthesis at subinhibitory concentrations and in a highly specific manner.

### MibEF are likely to be involved in immunity to microbisporicin in *M* *. corallina*

**Figure 7:**
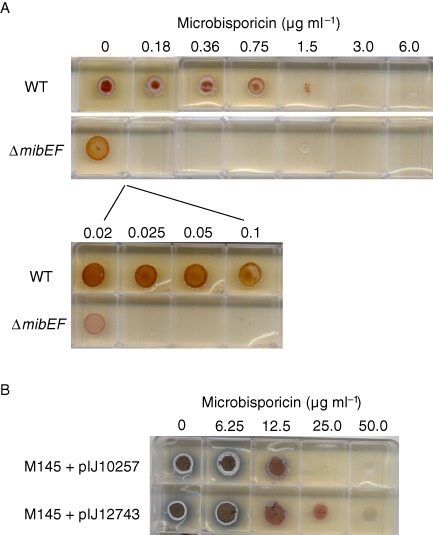
A. Wild‐type *M* *icrobispora corallina* (WT) and the *mib* *EF* mutant (Δ*mib* *EF*) were grown on MV01 agar medium containing increasing concentrations of microbisporicin. The wild‐type strain was able to grow readily at 0.75 μg ml^−1^ microbisporicin while the Δ*mib* *EF* mutant failed to grow at 0.025 μg ml^−1^ indicating an approximate 30‐fold increase in sensitivity to the compound. B. *S* *treptomyces coelicolor* M145 containing pIJ10257 (empty vector) or pIJ12743 (with *mib* *EF* expressed constitutively from *erm* E*p) were grown on R5 agar medium containing increasing concentrations of microbisporicin. Expression of *mib* *EF* resulted in an approximate twofold reduction in sensitivity to microbisporicin.

Previous bioinformatic analysis of the *mib* gene cluster identified three pairs of genes encoding ABC transporters: *mibTU*, *mibEF* and *mibYZ*. While deletion of *mibTU* had no apparent effect on microbisporicin production, the latter was essentially abolished in a *mibEF* mutant (Foulston and Bibb, 2010). To assess the possible role of MibEF in immunity, wild‐type *M. corallina* and the Δ*mibEF* mutant (M1131) were grown on V0.1 agar containing increasing concentrations of microbisporicin (Fig. 7A). The wild‐type strain grew well on 0.75 μg ml^−1^ of microbisporicin, whereas the Δ*mibEF* mutant failed to grow on 0.025 μg ml^−1^, exhibiting at least 30‐fold greater sensitivity towards the lantibiotic, suggesting a role for MibEF in immunity. Consistent with this, expression of *mibEF* from the constitutive ermE* promoter in pIJ12743 in *S. coelicolor* M145 led to a twofold increase in microbisporicin resistance (Fig. 7B) when compared with M145 containing the empty vector pIJ10257. Interestingly, homologues of MibZY, the transmembrane protein MibJ, and MibQ have also been implicated in immunity to microbisporicin in *Microbispora* sp. ATCC PTA5024 (Stegmann *et al*., 2014).

## Discussion

In this study, we demonstrate that RelA, and presumably ppGpp synthesis, can activate the complex regulatory pathway that leads to the biosynthesis of microbisporicin, a potent lantibiotic currently undergoing preclinical trials. Our model suggests that an immature and less active form of microbisporicin serves as the initial extracellular signalling molecule to coordinate production throughout the *M. corallina* population. This may reflect a mechanism adopted by the organism to protect itself and its siblings from the highly potent mature lantibiotic, ensuring expression of *mibTU* before committing to the production of microbisporicin. A similar feed‐forward regulatory mechanism has been proposed for the lantibiotic planosporicin (Sherwood and Bibb, 2013). Interestingly, in this case, planosporicin, which is considerably less active than microbisporicin (Sherwood and Bibb, 2013), serves as the specific signalling molecule.

Auto‐induction of antibiotic biosynthesis has also been observed in low‐GC Gram‐positive lantibiotic producing bacteria; examples include nisin (Kleerebezem, 2004), subtilin (Stein *et al*., 2002) and mersacidin (Schmitz *et al*., 2006), where at least some of the compounds have been suggested to function as quorum sensors that monitor population size and density (Stein *et al*., 2002; Kleerebezem, 2004). In contrast, in *M. corallina* and potentially other actinomycetes, auto‐induction is triggered by nutrient limitation which we propose serves to coordinate production of the antibiotic throughout the mycelial population, not all of which may be experiencing starvation, with the aim of achieving ecologically effective levels of the antibiotic.

We have shown that deletion of *mibEF* in *M. corallina* results in increased sensitivity to microbisporicin. Furthermore, heterologous over‐expression of *mibEF* in *S. coelicolor* resulted in an increase in resistance to the lantibiotic, suggesting that MibEF play a role in conferring immunity to microbisporicin in the natural producer. In previous work, deletion of *mibEF* essentially abolished microbisporicin production (Foulston and Bibb, 2010). The closest homologues of MibEF (with the exception of transporters encoded by closely related actinobacterial lantibiotic gene clusters, such as that for planosporicin; Sherwood and Bibb, 2013) are the immunity ABC transporters found in low‐GC Gram‐positive lantibiotic producers. This raises the intriguing possibility of the existence, at least for microbisporicin, of a fail‐safe mechanism that ensures that the production of a potent antibiotic does not take place unless the corresponding immunity system is in place. How this potential mechanism influences the expression of *mibR* and/or *mibX* and hence, microbisporicin biosynthesis remains to be determined.

Preclinical trials suggest that microbisporicin is a promising candidate for clinical development, which will require the provision of significant amounts of the purified compound. In addition to deciphering the complex regulatory mechanism that triggers microbisporicin production, we have demonstrated, by constitutively expressing the genes encoding the two transcriptional activators present in the *mib* gene cluster, how that knowledge can be used to markedly increase the productivity of the wild‐type strain, hopefully contributing to the future clinical use of this compound.

The experiments reported here on *M. corallina* were also carried out on the commercial producer of microbisporicin (NAI‐107) *Microbispora* sp. ATCC PTA5024 with essentially the same results. For continuity with previous work (Foulston and Bibb, 2010; 2011), only the *M. corallina* results are reported here.

## Experimental procedures

### Strains and general methods

The strains used and generated in this study are listed in Table 2. *E. coli* strains were grown and manipulated following standard methods (Sambrook *et al*., 1989; Gust *et al*., 2003). For conjugation, *M. corallina* NRRL 30420 spores were germinated for 21 h in 10 ml Difco Nutrient Broth (DNB, Becton Dickinson, Sparks, Maryland, USA), resuspended in 500 μl DNB, mixed with *E. coli* S17 (Simon *et al*., 1983) carrying the relevant conjugative and integrative vector and plated on Soya Flour Mannitol (SFM) agar containing 10mM MgCl_2_. After growth at 30°C for 20 h, the plates were overlaid with 100 μl of fosfomycin (100 mg ml^−1^) and the appropriate concentration of the antibiotic used to select for the vector. Plates were grown at 30°C until putative exconjugants were visible (3–5 weeks). Microbisporicin was detected as described previously (Foulston and Bibb, 2010). *S. coelicolor* strains were grown and manipulated as described previously (Kieser *et al*., 2000). Plasmids and oligonucleotides are described in Tables 1 and S1, respectively.

**Table 2:** Strains used and constructed in this study

Strain	Genotype	Reference
*Escherichia coli* DH5α	F^−^ φ80 *lacZ*ΔM15 Δ(*lacZYA*‐*argF*)U169 *recA*1 *endA*1 *hsdR*17 (r_k_^−^, m_k_^+^) *phoA supE*44 *thi*‐1 *gyrA*96 *relA*1 λ^−^)	Invitrogen™
*E. coli* ET12567	*dam‐13:: Tn9 dcm‐6 hsdM* CHL^R^, carrying helper plasmid pUZ8002	MacNeil *et al*., 1992
*E. coli* S17	*E. coli* strain carrying an integrated RP4 derivative	Simon *et al*., 1983
*Microbispora corallina* NRRL 30420	*M. corallina* wild‐type strain	Nakajima *et al*., 1999
*M. corallina* M1592	*M. corallina* + pIJ12572	This work
*M. corallina* M1593	*M. corallina* + pIJ12574	This work
*M. corallina* M1597	*M. corallina* + pIJ12572 + pIJ12574	This work
*M. corallina* M1596	*M. corallina* Δ*relA*	This work
*M. corallina* M1127	*M.corallina ΔmibA::aac(3)IV*	Foulston and Bibb, 2010
*M. corallina* M1131	*M.corallina ΔmibEF::aac(3)IV*	Foulston and Bibb, 2010
*Micrococcus luteus* ATCC 4698	Bioassay indicator microorganism	ATCC
*Streptomyces coelicolor* M145	*S. coelicolor* A3(2) plasmid‐free derivative	Kieser *et al*., 2000
*S. coelicolor* M145 + pIJ12743	M145 + pIJ12743	This work
*S. coelicolor* M145 + pIJ12586	M145 + pIJ12586	This work
*S. coelicolor* M571	*S.coelicolor* M145 Δ*relA*	R. Chakraburtty and M. J. Bibb, unpublished
*S. coelicolor* M571 + pIJ12586	M571 + pIJ12586	This work
*S. coelicolor* M1152	M145 derivative Δ*act* Δ*red* Δ*cpk Δcda rpoB*(C1298T)	Gomez‐Escribano and Bibb, 2011
*S. coelicolor* M1598	M1152 + pIJ10257	This work
*S. coelicolor* M1594	M1152 + pIJ12576	This work
*S. coelicolor* M1595	M1152 + pIJ12574	This work
*S. coelicolor* M1594 derivatives		
	M1594 + pIJ12579	This work
	M1594 + pIJ12580	This work
	M1594 + pIJ12581	This work
	M1594 + pIJ12582	This work
	M1594 + pIJ12583	This work
	M1594 + pIJ12584	This work
*S. coelicolor* M1595 derivatives		
	M1595 + pIJ12579	This work
	M1595 + pIJ12580	This work
	M1595 + pIJ12581	This work
	M1595 + pIJ12582	This work
	M1595 + pIJ12583	This work
	M1595 + pIJ12584	This work

CHL, chloramphenicol.

### Construction of a *relA* mutant of *M* *. corallina*

Chromosomal regions flanking the *relA* coding sequence (Sosio *et al*., 2014) were amplified by PCR and cloned into pIJ12738 (Fernández‐Martínez and Bibb, 2014). The 5′ flanking region was amplified to generate a 1902 bp fragment with terminal *Nco*I and *EcoR*I sites while the 3′ region was amplified to generate a 1945 bp fragment with terminal *Eco*RI and *Xba*I sites. These two fragments were cloned into pIJ12738 digested with *Nco*I and *Xba*I to generate pIJ12749 with the I‐SceI site adjacent to the introduced fragments. pIJ12749 was then introduced into *E. coli* S17 by transformation. Conjugation between the *E. coli* S17 derivative and *M. corallina* was carried out as described earlier. Chromosomal integration of pIJ12749, confirmed by PCR analysis (data not shown), generated *M. corallina ΔrelA*_int, which still contained *relA*. A 806 bp *Nde*I‐*Sac*II fragment containing the I‐SceI Meganuclease gene codon‐optimised for expression in actinomycetes was excised from pIJ12739 (Fernández‐Martínez and Bibb, 2014) and cloned into pIJ10257 to generate pIJ12750, which was then conjugated into *M. corallina ΔrelA*_int to induce a double‐strand break at the introduced I‐SceI site. Three individual exconjugants were streaked on SFM agar containing hygromycin (10 μg ml^−1^) and grown at 30°C until sporulation. PCR analysis showed that one of these exconjugants, M1596, lacked the chromosomal *relA* sequence (data not shown).

### *gusA* transcriptional fusions

Promoter regions from the *mib* gene cluster (P*_mibJ_* 299bp, P*_mibQ_* 166 bp, P*_mibR_* 384 bp, P*_mibX_* 254 bp, P*_mibA_* 244 bp and P*_mibE_* 440 bp) were amplified using oligonucleotides with 5′ *Xba*I and 3′ *Kpn*I sites, confirmed by nucleotide sequencing and ligated into pGUS (Myronovskyi *et al*., 2011) digested with the same enzymes to generate the following plasmids containing *gusA* under the control of each of the promoters: pIJ12579 (P*_mibA_*‐*gusA*), pIJ12580 (P*_mibE_*‐*gusA*), pIJ12581 (P*_mibJ_*‐*gusA*), pIJ12582 (P*_mibQ_*‐*gusA*), pIJ12583 (P*_mibR_*‐*gusA*) and pIJ12584 (P*_mibX_*‐*gusA*). The plasmids were integrated at the ΦC31 *attB* site of *S. coelicolor* M1152 (Gomez‐Escribano and Bibb, 2011) after conjugation and at the same site in *S. coelicolor* M1594 and M1595, strains carrying constructs based on pIJ10257 (Hong *et al*., 2005) in which *mibR* or *mibX*, respectively, were expressed constitutively from ermE*p. Exconjugants were selected using apramycin (25 μg ml^−1^) and confirmed by PCR.

### β‐Glucuronidase assays

Spectrophotometric β‐glucuronidase assays were carried out as described previously (Sherwood and Bibb, 2013). Enzymatic activity was plotted as Miller units calculated as 1000 × (OD420 of sample − OD420 of blank)/[time of reaction in minutes × volume of culture assayed (in ml)] and expressed per milligram of protein.

### Constitutive expression of *mibR* and *mibX* in *M* *. corallina* and *S* *. coelicolor* M1152

PCR products containing *mibR* or *mibX* (extending from start to stop codons) were generated by high‐fidelity PCR using the primers listed in Table S1 and confirmed by nucleotide sequencing. The *mibR* fragment was cloned into the integrative vector pIJ8600 (Takano *et al*., 1995) to fuse *mibR* to the inducible *tipA* promoter generating pIJ12572, which was introduced into *M. corallina* by conjugation. For introduction into *S. coelicolor* M1152, the same *mibR* fragment was cloned into the integrative vector pIJ10257 to fuse *mibR* to the constitutive ermE* promoter generating pIJ12576. Similarly, the *mibX* fragment was also cloned into the integrative vector pIJ10257 to fuse *mibX* to the constitutive ermE* promoter generating pIJ12574. These constructs were transferred into *M. corallina* by conjugation from *E. coli* S17 and into *S. coelicolor* M1152 by conjugation from *E. coli* ET12567/pUZ8002 as described previously (Kieser *et al*., 2000).

### Expression of *mibEF* in *S* *. coelicolor* M145

A PCR product containing *mibEF* (extending from the start codon of *mibE* to the stop codon of *mibF*) was generated by high‐fidelity PCR using the primers listed in Table S1 and confirmed by nucleotide sequencing (data not shown). This fragment was cloned into the integrative vector pIJ10257 to fuse *mibEF* to the constitutive ermE* promoter generating pIJ12743. This plasmid was transferred into *S. coelicolor* M145 by conjugation from *E. coli* ET12567/pUZ8002 as described previously (Kieser *et al*., 2000).

### RT‐PCR analysis

The *M. corallina* wild‐type strain was grown in 10 ml VSP liquid medium (Marcone *et al*., 2010) until an OD_600_ of 0.4 was reached (after 3–4 days of growth) and then 1 ml of this culture was transferred to 50 ml VSP liquid medium (designated time 0). For nested RT‐PCR analysis of the *mibR* promoter (Fig. 2), RNA was extracted from the mycelia from 4 ml of culture sampled after 16, 24, 40 and 48 h of growth from time 0 (Tunca *et al*., 2007). For RT‐PCR analysis of the effect of deletion of *relA* on *mib* gene expression, *M. corallina* wild‐type and the *relA* mutant strain were grown in 10 ml SMM medium (Kieser *et al*., 2000) to an OD_600_ of 0.4 (reached after 6–7 days of growth) and then 1 ml of this culture was transferred to 50 ml SMM liquid medium (designated time 0). RNA was extracted from the mycelia from 4 ml of culture sampled after 48 h of growth from time 0 (Tunca *et al*., 2007). Mycelial pellets were resuspended in 1 ml RTL buffer with lysing matrix B (MP Biomedicals, Solon, Ohio, USA) and homogenised using a Camlab Omni Bead Ruptor 24 Drive Unit (Camlab, Cambridge, UK). Two pulses of 30 s of intensity 6.0 were applied with cooling down for 1 min on ice between pulses. Supernatants were centrifuged for 10 min at 13,000 r.p.m. and then treated according to the instructions given in the RNA Easy Kit (Qiagen, Crawley, UK). The RNA samples were treated with DNase I (Invitrogen) until they were free of DNA contamination (determined by PCR amplification). RNA was quantified and equal amounts from each sample were converted to cDNA following the manufacturer's instructions (SuperScript®, Invitrogen). Oligonucleotide pairs listed in Table S1 were used to amplify nested fragments of the *mibR* promoter region. The oligonucleotide pairs used to analyse expression of the *mib* cluster in the wild‐type and *relA* mutant strains were described previously (Foulston and Bibb, 2010). Amplification was also attempted using the same oligonucleotide pairs on RNA samples that had not been treated with reverse transcriptase to confirm lack of DNA contamination.

### RACE

The 5′ end of the *mibR*p2 transcript was identified by using a 5′ RACE (rapid amplification of cDNA ends) kit (Invitrogen, Paisley, UK, version 2.0) following the manufacturer's instructions. Briefly, first‐strand cDNA synthesis was carried out using 5 μg of RNA, reverse transcriptase and the oligonucleotide primer RACE_mibR_R1 (Table S1). cDNA was purified using the SNAP columns provided in the kit, and poly(dC) tails were added to the 3′ ends using terminal deoxynucleotidyl transferase. PCR amplification of the tailed cDNA was carried out initially using the 5′ RACE abridged anchor primer with the first‐strand primer RACE_mibR_R1 (Table S1). A dilution of the PCR mixture was then subjected to a second amplification using the abridged anchor primer with the second nested primer RACE_mibR_R2 (Table S1). The PCR product was gel‐purified and a portion sequenced directly using oligonucleotide RACE_mibR_R2 as primer (RACE1 in Fig. S1B). Another portion was used for cloning into pGEM‐TEasy (Promega UK, Southampton, UK) and the cloned PCR fragment sequenced using RACE_mibR_R2 as primer (RACE2 in Fig. S1B).

### Induction of microbisporicin production in *M* *. corallina*

To test for induction of microbisporicin production in wild‐type *M. corallina*, either *M. corallina* M1592 (*mibR* expression strain) or 2.5 μg of purified microbisporicin (in 10% DMSO) dried on a filter paper disc were placed on V0.1 (Marcone *et al*., 2010) agar plates adjacent to streaks of wild‐type *M. corallina* and a non‐producing mutant [Δ*mibA*, (Foulston and Bibb, 2010) ] as a control; the plates were overlaid with *Micrococcus luteus* after incubation at 30°C for 5 days.

## Supporting information

Supporting information
