# Supplementary material for: A rel A‐dependent regulatory cascade for auto‐induction of microbisporicin production in M icrobispora corallina
Source: Mol Microbiol. 2015 May 29;97(3):502–14. doi: 10.1111/mmi.13046 (PMC4973701; doi:10.1111/mmi.13046)
Supplement: Supplementary file 1 — Supporting information [file MMI-97-502-s001.docx]

**A *relA*-dependent regulatory cascade for auto-induction of microbisporicin production in *Microbispora corallina* – Supplementary Information**

Lorena T. Fernández-Martínez, Juan P. Gomez-Escribano and Mervyn J. Bibb*

Department of Molecular Microbiology, John Innes Centre, Norwich Research Park, Norwich, NR4 7UH, UK

Running title: Regulation of microbisporicin biosynthesis in *M. corallina*

*For correspondence. E-mail [mervyn.bibb@jic.ac.uk](mailto:mervyn.bibb@jic.ac.uk); Tel. (+44) 1603 450776

Keywords: lantibiotic, sigma factor, ppGpp, actinomycete, signalling molecule

**Supplementary Information**

**Table S1.** Oligonucleotides used in this study.

| **Oligonucleotide** | **Sequence** | **Notes** |
| --- | --- | --- |
| mibAp_XbaI_F | AATCTAGATCATGAGGACGGCATCCT | Amplification of the *mibA-V* promoter region for pGUS fusion |
| mibAp_KpnI_R | AAGGTACCCATTGCTCGCTCCTTTCC | Amplification of the *mibA-V* promoter region for pGUS fusion |
| mibEp_XbaI_F | AATCTAGAACGGACGGCGCCGATGGC | Amplification of the *mibE-N* promoter region for pGUS fusion |
| mibEp_KpnI_R | AAGGTACCGATATCAGGGCCGCCATCGAC | Amplification of the *mibE-N* promoter region for pGUS fusion |
| mibJp_XbaI_F | AATCTAGAGCGGTTCCGGCGCCTCAGG | Amplification of the *mibJ-O* promoter region for pGUS fusion |
| mibJp_KpnI_R | AAGGTACCGACCATCTCCACACCAGGG | Amplification of the *mibJ-O* promoter region for pGUS fusion |
| mibQp_XbaI_F | AATCTAGAAGGGTCGCCCTTCGAGAACTG | Amplification of the *mibQ* promoter region for pGUS fusion |
| mibQp_KpnI_R | AAGGTACCGTCGTGTTCGTCATGCCC | Amplification of the *mibQ* promoter region for pGUS fusion |
| mibRp_XbaI_F | AATCTAGACAAGGGCAGCGGTGGCGG | Amplification of the *mibR* promoter region for pGUS fusion |
| mibRp_KpnI_R | AAGGTACCCCTTTGCCGGTCGGGCAC | Amplification of the *mibR* promoter region for pGUS fusion |
| mibXp_XbaI_F | AATCTAGACATTGCTCGCTCCTTTCC | Amplification of the *mibXW* promoter region for pGUS fusion |
| mibXp_KpnI_R | AAGGTACCTCATGAGGACGGCATCCT | Amplification of the *mibXW* promoter region for pGUS fusion |
| mibRp2_XbaI_F | AATCTAGAGAACACGACCAGAGCCCGCC | Amplification of the *mibRp2* promoter for pGUS fusion |
| mibRp2_KpnI_R | AAGGTACCACGCAGGAGCCGGATTTTCC | Amplification of the *mibRp2* promoter for pGUS fusion |
| mibX_NdeI_F | AAATATACATATGATGAGACGCGTGGCCGACGGCG | Amplification of *mibX* for constitutive expression |
| mibX_HindIII_R | AAAAGCTTTCATCTATCACCAGTCCGCCCC | Amplification of *mibX* for constitutive expression |
| mibR_NdeI_F | AAATATACATATGGTGCCCGACCGGCAAAGGAC | Amplification of *mibR* for constitutive expression |
| mibR_HindIII_R | AAAAGCTTTCAGCACGACAAACCGCCC | Amplification of *mibR* for constitutive expression |
| mibEF1 | CATATGGCGGCCCTGATATCCAC | Amplification of *mibEF* for constitutive expression |
| mibEF2 | AAGCTTCCGTTCGTGGGTCAGG | Amplification of *mibEF* for constitutive expression |
| mibRpECF_F | GAAACTGCCGGAGGTAACAG | Corresponds to RT-PCR forward oligo ECF in Fig.2 |
| mibRp2_1_F | TCCGAAAACTTACCCGAGTG | Corresponds to RT-PCR forward oligo P1 in Fig.2 |
| mibRp2_2_F | ACGACCTACGCTCTCAACGA | Corresponds to RT-PCR forward oligo P2 in Fig.2 |
| mibRp2_3_F | GAGCAACAAGCAGGACATCA | Corresponds to RT-PCR forward oligo P3 in Fig.2 |
| mibRp2_4_F | GACGAACACGACCAGAGCCC | Corresponds to RT-PCR forward oligo P4 in Fig.2 |
| mibRp_R | GTCGCTCTCCTCGTCCTTT | Corresponds to RT-PCR reverse oligo R common to all reactions in Fig.2 |
| RACE_mibR_R1 | CCGGTTGTGCCGCACCGCAC | RACE first strand primer |
| RACE_mibR_R2 | CGCGCACCATCGGCATCGCG | RACE second nested primer |
| relA_left-arm_F_NcoI | AAAACCATGGGACCATGACCATCAAGACGA | Amplification of the *relA* 5' flanking region |
| relA_left-arm_R_EcoRI | AATTAGAATTCGTCCGCGGTCTGTGAATG | Amplification of the *relA* 5' flanking region |
| relA_right-arm_F_EcoRI | AATTAGAATTCTCCTCAGTGTATCCGTGCTG | Amplification of the *relA* 3' flanking region |
| relA_right-arm_R_XbaI | AATCTAGAAGGTCAGCTCCCAGGTCAT | Amplification of the *relA* 3' flanking region |

**Figure S1.**

A.


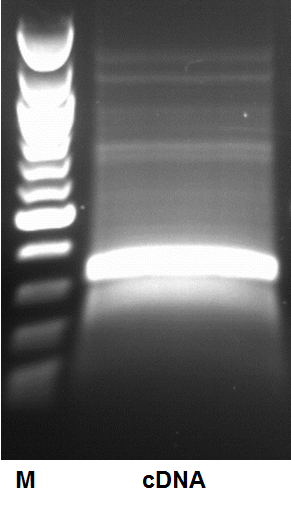


B.


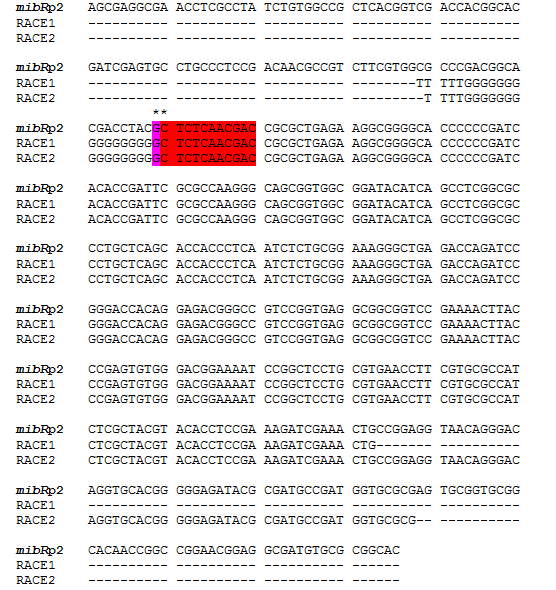


**Figure S1.** A. PCR amplification of the C-tailed cDNA using the abridged anchor primer and the second nested gene specific primer (RACE_mibR_R2). The PCR product was gel-purified and sequenced directly using the same RACE_mibR_R2 primer; a sample was also cloned in pGEM-TEasy (Promega) and sequenced again using the RACE_mibR_R2 primer. B. Alignment of the *mibR*p2 region with sequences obtained directly from the purified PCR product (RACE 1) and after cloning (RACE 2). The likely transcriptional start site(s) is (are) indicated by asterisks (the homopolymeric run of Cs added after cDNA synthesis does not allow us to determine whether transcription initiates at the G, C or both of the nucleotides).
